# Evidence from high‐income countries on the effectiveness of psychosocial interventions to improve mental health, wellbeing and quality of life for adults living with HIV: a systematic review and meta‐analysis

**DOI:** 10.1002/jia2.26424

**Published:** 2025-03-26

**Authors:** Ada R. Miltz, Janey Sewell, Fumiyo Nakagawa, Sophia M. Rein, Lorraine Sherr, Alison Rodger, Andrew N. Phillips, Sanne vanLuenen, Nadia Garnefski, Vivian Kraaij, Colette J. Smith, Valentina Cambiano, Fiona C. Lampe

**Affiliations:** ^1^ Institute for Global Health University College London London UK; ^2^ Royal Free London NHS Foundation Trust London UK; ^3^ Section of Clinical Psychology, Institute of Psychology, Faculty of Social and Behavioural Sciences Leiden University Leiden The Netherlands

**Keywords:** anxiety, depression, HIV, intervention, psychosocial, RCT

## Abstract

**Introduction:**

There is a need to synthesize recent evidence on the effectiveness of psychosocial interventions to improve mental health, quality of life and wellbeing in adults living with HIV in high‐income countries. A systematic review and meta‐analysis was conducted to address this research gap.

**Methods:**

Medline, Embase, Psychinfo and Web of science were searched (from 2008 to December 2023). In total, 67 randomized controlled trials (RCTs) of psychosocial intervention among adults living with HIV in high‐income countries were eligible.

**Results:**

In the meta‐analysis, there was an overall positive effect of interventions on reducing depression (*N* = 40; standardized mean difference [SMD] −0.19 [95% CI: −0.29, −0.10]), anxiety (*N* = 15; SMD −0.12 [−0.23, −0.02]), stress (*N* = 13; SMD −0.22 [−0.41, −0.04]), and other measures of poor wellbeing (*N* = 19; SMD −0.18 [−0.35, −0.02]) and increasing levels of coping/self‐efficacy (*N* = 8; SMD 0.17 [0.04, 0.31]). For depression, interventions that used symptom screening above a threshold score to identify eligible individuals were more effective than those without such an eligibility criterion (SMD −0.29 vs. ‐0.10, *p* = 0.023). Interventions compared to standard care controls had a greater effect on depression versus interventions compared to not standard care controls, when the latter category included standard care controls that received intentional support (SMD ‐0.28 vs. ‐0.11, *p* = 0.035). There was also weak evidence of an overall positive effect on: reducing stigma (*N* = 7; SMD −0.17 [−0.35, 0.02]), and improving social support/participation (*N* = 6; SMD 0.17 [−0.02, 0.35]), mental health quality of life (*N* = 12; SMD 0.09 [−0.01, 0.19]), physical health quality of life (*N* = 11; SMD 0.07 [−0.02, 0.16]) and quality of social life (*N* = 6; SMD 0.10 [−0.04, 0.24]). There was no evidence found for an effect on loneliness, although data were limited.

**Discussion:**

Pooled effect estimates were small or small tomoderate. In line with previous literature, there was no evidence of differential effects on depression according to the intervention type (psychotherapeutic vs. other).

**Conclusions:**

Evidence from RCTs suggest that psychosocial interventions are effective in improving mental health for adults living with HIV in high‐income settings. Interventions were more effective at reducing depression when targeted at those screening positive for mental health symptoms and when compared to a standard care only control group. There was some evidence that longer, more intensive interventions were more effective.

## 1 INTRODUCTION

The landscape of HIV treatment and prevention has changed dramatically in the past decades with the development of highly effective and tolerable antiretroviral treatments (ARTs) [1]. Improvements in virological and clinical outcomes of treatment and knowledge that having an undetectable viral load indicates that HIV cannot be transmitted sexually [2], may have a positive impact on the mental health and wellbeing of people living with HIV. However, HIV‐related stigma and discrimination persists and reinforces existing social inequalities in gender, ethnicity and sexuality [3, 4]. Poor mental health, in particular symptoms of depression, remains prevalent among people living with HIV. In a review of depression in people living with HIV in 2019 [5], the prevalence of current depression as measured via symptom questionnaire ranged from 6% to 41% (median of 24.6%) across 22 studies. Lifetime major depressive disorder as measured via structural clinical interviews ranged from 26% to 50% (median of 42%) across five studies. Depression negatively impacts ART adherence, therapeutic and clinical outcomes, and may also play a role in substance use and sexual behaviour [6−10]. In many people living with HIV, provision of effective psychosocial support may be key to improving health and wellbeing outcomes.

Numerous psychosocial interventions that aim to change negative thought patterns, emotions and/or behaviours, and which could potentially impact on mental health outcomes, have been developed and evaluated among people living with HIV. A previous systematic review and meta‐analysis investigated the overall effectiveness of psychosocial interventions for people living with HIV, across all countries, for randomized controlled trials (RCTs) published from 1996 to 2014 [11]. The authors found an overall small positive effect when combining 62 studies across measures of depression, anxiety, quality of life and general psychological wellbeing. Studies were conducted in the United States, Canada China, Iran, Kenya, Nigeria, South Africa, Switzerland, Tanzania, Thailand, The Netherlands, Uganda,Vietnam, Mexico, South Africa and Puerto Rico.

To better understand the effectiveness of psychosocial interventions in a high‐income setting, there is a need to synthesize recent evidence from trials in high‐income countries. The aims of this systematic review and meta‐analysis were to: (i) investigate the overall effectiveness of psychosocial interventions to reduce symptoms of depression and anxiety and improve quality of life and psychological wellbeing in adults living with HIV in high‐income countries, and (ii) investigate whether important characteristics of an intervention or trial (type, provider and length of intervention, control group comparator, mental health inclusion criteria, study quality) may influence effectiveness.

## 2 METHODS

### 2.1 Study selection

**Figure 1:**
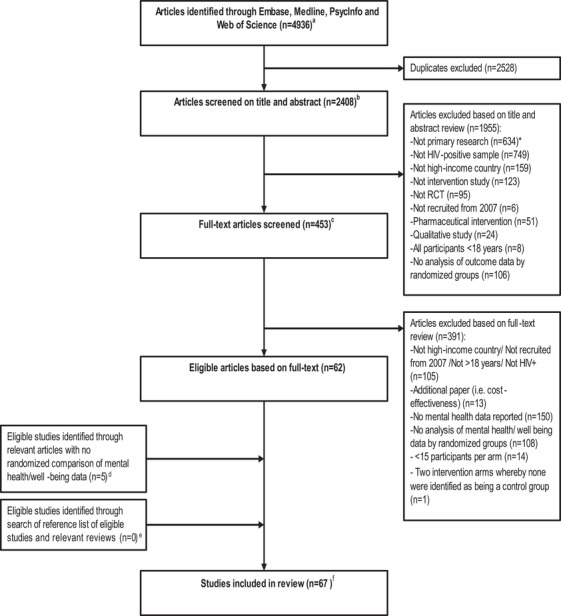
Flow chart of study inclusion and exclusion. ^a^A systematic search was undertaken of the electronic databases Embase, Medline, PsycInfo and Web of Science to identify RCTs of psychosocial interventions for people living with HIV in high‐income countries published from 2008 up to 14th December 2023. Search terms included those related to: (a) HIV, (b) psychosocial interventions (definition based on a previous review [11], e.g. psychotherapy, counselling, coaching, social support, Cognitive behavioural therapy (CBT), meditation), (c) RCTs (based on a Cochrane recommended search strategy [45]) and (d) high‐income countries (based on World Bank Data [46, 47]). Terms related to mental health/wellbeing/quality of life outcomes were not specified in the search strategy. This was to identify studies that did not mention these outcomes in the title, abstract or key words but did report on this data in the paper. Search terms specifically related to studies of people without HIV (e.g. HIV self‐testing, PrEP, PEP) and pharmaceutical interventions (e.g. pharmacokinetic, immunogenicity) were used to remove ineligible studies. ^b^Stage 1: The title and abstract of all identified studies was reviewed for eligibility. Two reviewers (ARM and JS) independently assessed a sample of 100 studies (first and last 50 studies when sorted by author name). Thereafter, one reviewer (ARM) completed the abstract review. ^c^Stage 2: Two reviewers (ARM and/or JS) reviewed the full‐text of all studies selected in stage 1. Disagreement about study selection was resolved via discussion with one or more co‐authors. Of note, studies were included regardless of whether mental health, quality of life or wellbeing measures collected were the primary endpoint of the trial. ^d^Authors of recently published papers (2019−2023) were contacted. Forty‐six authors were contacted, of whom 27 responded, and five provided data. ^e^Title and abstract of all articles referenced in 67 eligible studies were imported into Endnote (*N* = 2499). All references with “HIV” in the title that were published after 2007 were reviewed (*N* = 804). Of these, 37 were deemed to be potentially eligible and the full‐text articles were reviewed. No articles were found to be eligible. Articles were excluded for the following reasons: not HIV‐positive sample (*n* = 2), not high‐income country (*n* = 1), not RCT (*n* = 8), not from 2007 onwards (*n* = 10), not >18 years of age (*n* = 2), no randomized comparison (*n* = 3), no mental health/wellbeing data collected (*n* = 5), and six articles were based on an eligible study already captured in the systematic review. Previous reviews and meta‐analyses on this subject were also searched to identify eligible studies—no eligible studies that were not already captured in the review were identified. ^f^A protocol to extract the data from eligible studies was developed [14, 48]. One reviewer (ARM, SMR or JS) initially extracted the data from each study. A second blinded reviewer checked for data extraction errors (FCL, VC, FN). Authors were contacted to retrieve information that was not published.

A systematic search was undertaken to identify RCTs of psychosocial interventions for adults living with HIV (Figure 1). The healthcare infrastructure may differ between high and middle/low‐income settings, and mental health facilities and services may also differ, with implications for the treatment and prevalence of mental health conditions. In view of this, this review was restricted to studies conducted in high‐income countries. Studies published from 2008 up to 14th December 2023 were reviewed.

Studies were included in the current review if they met all the following criteria: (i) participants living with HIV (aged 18 years or older); (ii) intervention that included either a psychosocial component, or non‐pharmaceutical component that aimed to change thoughts, emotions and/or behaviour; (iii) RCT; (iv) data collected on mental health, quality of life or wellbeing measures, including social support and HIV‐related stigma (by validated symptom questionnaire, professional diagnosis or treatment, or self‐rated measure); (v) data from high‐income country; (vi) recruitment of participants from 2007 onwards (to identify interventions conducted in the modern ART era); and (vii) ≥15 individuals randomized and analysed per study arm. Studies identified by the search that included some participants aged under 18 years were included in the review provided that the average age of participants was over 18 years.

The title and abstract of all identified studies were reviewed for eligibility. Two reviewers (ARM and JS) reviewed the full‐text of all selected studies. Of note, studies were included regardless of whether mental health, quality of life or wellbeing measures collected were the primary endpoint of the trial. The search strategy, study selection and data extraction are described in further detail in footnotes ^a−f^ under Figure 1.

### 2.2 Classification of psychosocial interventions

Each intervention was classified as psychotherapeutic, supportive, general relaxation or physical exercise as defined in Table 1. Interventions that fit into multiple categories were classified either as “multiple including psychotherapeutic” or “multiple not including psychotherapeutic.”

**Table 1:** Classification of intervention types^a^

Intervention types	Description
Psychotherapeutic	Specific psychological techniques (e.g. CBT, other talk therapies, motivational interviewing)
Supportive	Help from others without specific psychological technique (e.g. social support, information, psychoeducation, financial support, risk‐reduction, general counselling)
General relaxation	Mindfulness, meditation, yoga
Physical exercise	Physical activity
Multiple including psychotherapeutic	More than one intervention type including specific psychological techniques
Multiple not including psychotherapeutic	More than one intervention type not including specific psychological techniques

Abbreviation: CBT, Cognitive behavioural therapy.

^a^ Classifications were derived based on a previous review of psychosocial interventions [11].

### 2.3 Classification of control groups

Control groups were classified as standard care, standard care with waitlist, enhanced standard care or active control groups. All control groups that were described by authors as standard care were included in the first category; however, there was a sub‐set that were judged in this review to have included a component that may be additional to standard care. For instance, standard care with a letter to the participants’ primary caregiver, regular phone calls and counselling. This sub‐group of “standard care” is referred to as “standard care with intentional support” in this review and is investigated in sensitivity analyses as described in the Statistical methods section below.

### 2.4 Classification of outcome measures

Data were extracted on all mental health, wellbeing and quality of life measures (Table 2). These outcome measures were grouped into one of nine categories: (i) depression; (ii) anxiety; (iii) stigma; (iv) stress; (v) loneliness; (vi) coping/self‐efficacy; (vii) social support/participation; (viii) quality of life; and (ix) other wellbeing measures. For categories (i)−(v), higher scores relate to higher symptomatology/poorer wellbeing. For categories (vi)−(viii), higher scores relate to lower symptomatology/greater wellbeing. In the latter category (ix), the direction of scores was made consistent such that higher scores on questionnaires related to higher symptomatology, that is poorer wellbeing. For some measures, this necessitated reversing data from the intervention and control arms to calculate effect estimates that fit the interpretation of outcomes in this category.

**Table 2:** Classification of outcome measures

Outcome category	Questionnaire scales
Depression	Center for Epidemiological Studies‐Depression (CES‐D); Beck's Depression Inventory (BDI); Patient Health Questionnaire (PHQ); Depression Anxiety Stress Scales (DASS); Geriatric Depression Scale (GDS); Hospital Anxiety and Depression Scale (HADS); Hamilton Depression Rating Scale (HAM‐D); Hopkins Symptom Checklist (HSC SCL); Montgomery‐Asberg Depression Rating Scale (MADRS); PROMIS Depression Scale (PROMIS) Quick Inventory of Depressive Symptomatology (QIDS); Mental Health Inventory Survey (MHI); Profile of Mood States (POMS)
Anxiety	General Anxiety Disorder (GAD‐7); Beck Anxiety Inventory (BAI); The State‐Trait Anxiety Inventory (STAI); Hospital Anxiety and Depression Scale (HADS); Mental Health Inventory Survey (MHI); PROMIS Anxiety Scale (PROMIS); Depression Anxiety Stress Scales (DASS); Structured Interview Guide for the Hamilton Anxiety scale (SIGH‐A); HIV health‐related anxiety; Patient‐Reported Outcomes Measurement Information System
Stigma	Internalized HIV/AIDS‐Related Stigma Scale (IHSS); HIV Stigma Scale (HSS); Stigma Scale for Chronic Illnesses (SSCI); Earnshaw and Chaudoir HIV stigma mechanisms
Stress	Perceived Stress Scale (PSS); Depression Anxiety Stress Scales (DASS); Life Experiences Survey (LES)^a^; HIV‐related Life‐Stressor Burden questionnaire; Parenting Stress Index‐Short Form (PSI‐SF)
Loneliness	Modified‐UCLA Loneliness Scale; 3‐Item Loneliness (3IL) Scale
Coping/self‐efficacy	Coping Self‐Efficacy Scale (CSES); Brief COPE; Patient Activation Measure (PAM); Distress Tolerance Scale (DTS); Health education impact questionnaire (HeiQ); Positive outlook self‐efficacy scale (POSE)^b^; Silencing the Self Scale (STSS)^c^; Brief resilience scale
Social support/participation	Social Provisions Scale (SPS); Medical Outcomes Study (MOS); Provision of Social Relations Scale (PSRS); Health education impact questionnaire (HeiQ); Inventory of Interpersonal Problems (IIPs)^d^; Multidimensional Scale of Perceived Social Support (MSPSS); Patient‐Reported Outcomes Measurement Information System (PROMIS); Positive outlook self‐efficacy scale (POSE)^e^
Quality of life	Medical Outcomes Study‐HIV (MOS SF‐12, SF‐36); EuroQol 5 Dimensions Index (EQ‐5D); HIV/AIDS Targeted Quality of Life Instrument (HAT‐QoL); Multidimensional Student's Life Satisfaction Scale (MSLSS); Patient‐Reported Outcome Quality of Life‐HIV (PROQOL‐HIV); Health‐related quality of life (WHOQOL‐HIV)
Other wellbeing measures	Differential Emotions Scale (DES); Clinical Global Impression (CGI); Brief Symptom Inventory (BSI); Health Education Impact Questionnaire (HeiQ); Mental Health Inventory (MHI); Beck Hopelessness Scale (BHS); Five Facet Mindfulness Questionnaire (FFMQ); HIV Symptom Distress Scale (SDS); Mindful Attention and Awareness scale (MAAS); Nottingham Health Profile (NHP); Positive and Negative Affect Schedule (PANAS); Positive Outlook Self‐Efficacy Scale (POSE)^f^; Profile in Mood States (POMS); Quality of Wellbeing (QWB); Rosenberg Self‐Esteem Scale (RSES); Satisfaction with Life Scale (SWLS); Subjective Wellbeing (SWB); Symptom Distress Scale (SDS); Warwick‐Edinburgh Mental Well‐Being Scale (WEMWBS)

Of note, the same questionnaire may appear in multiple outcome categories as different subscales were used to measure the various conditions/symptoms.

^a^ Modified to include only those events which were moderately to severely stressful for people living with HIV.

^b^ POSE subscales concerning relationships, knowledge and communication.

^c^ Silencing the self (STS): higher scores = greater silencing of self, therefore, the mean (SD) scores for intervention and control arms were reversed so that a positive effect estimate reflects that the intervention improved the outcome, that is less silencing of self, to be consistent with other measures in this category.

^d^ Higher scores = more interpersonal difficulties.

^e^ POSE subscale concerning social participation.

^f^ POSE subscale concerning emotions/emotional distress.

### 2.5 Risk of bias

Twelve criteria for assessing the level of possible risk of bias were developed based on the Cochrane Collaboration's Tool for assessing risk of bias [12] and a review about defining empirically supported psychological treatments [13]. Criteria consisted of:

- Random allocation to arms (yes, no, not mentioned)
- Concealment of allocation (yes, no, not mentioned)
- Power calculation (yes, no, not mentioned)
- Treatment manual (yes, no, not mentioned)
- Provider training (yes, no, not mentioned)
- Provider protocol adherence checked (mentioned and achieved, mentioned, not mentioned)
- Intention‐to‐treat analysis, meaning participants were analysed as part of the study arm they were randomized to at baseline (yes, no)
- 30% or less loss‐to‐follow‐up in both arms at the initial follow‐up time point (yes, no)
- Attrition bias considered (yes [assessed characteristics of participants lost vs. not lost to follow‐up, imputation methods for missing data, <10% loss‐to‐follow‐up in both arms], somewhat [discussed only], no [no discussion])
- Appropriate measurement of the outcome (yes, no [i.e. not validated questionnaire])
- Outcome measurement was the same in both arms (yes, no)
- No evidence of selective reporting (yes, no [results selected on the basis of multiple eligible outcomes, e.g. scales, definitions, time points or multiple analyses])

Studies that received a judgement of “yes” scored 1 and studies that received a judgement of “no” or “not mentioned” scored 0. On criterion (vi), studies that received a judgement of “mentioned and achieved” scored 1, studies that received a judgement of “mentioned” scored 0.5, and studies that received a judgement of “not mentioned” scored 0. On criterion (ix), studies that received a judgement of “somewhat” scored 0.5, and judgement of “yes” scored 1 and “no” scored 0. Computer and self‐help interventions were not judged on criteria (iv)−(vi). Scores were added up for each study and the percentage of eligible criteria met was calculated. Studies with 0–49% of criteria met were considered to be of lower quality and, therefore, higher risk of bias, 50–74% of medium quality, and 75% plus of higher quality.

### 2.6 Statistical methods

Most studies reported results in terms of mean scores in the relevant outcomes for intervention and control groups, Therefore, Cohen's *d* was used to produce a standardized mean difference (SMD) and 95% confidence interval (CI) comparing intervention with control group (Cohen's *d* =$\frac{{\bar{x}}_{1}-{\bar{x}}_{2}}{s_{P}}$; difference between two sample means (mean in intervention group minus mean in control group)/pooled standard deviation) for each relevant outcome, for each trial. Separate meta‐analyses were conducted for each of the nine outcome measure categories defined above. Therefore, each trial could contribute to multiple outcome categories. If an individual trial used more than one outcome measure from a specific category, then the outcome that was more frequently used across the reviewed studies was included and the others were ignored. For instance, if a study used the BDI and the PHQ‐9 questionnaires to measure depression, then the BDI was used as it was reported more frequently by the studies overall. In this way, no study was included more than once in any specific meta‐analysis. For all outcomes, data were included from the follow‐up time point closest to 3 months if there was more than one follow‐up in a study.

Six forest plots of findings from meta‐analyses are presented. Depression and anxiety outcome measures are presented separately, grouped by the intervention classification. The remaining outcomes are presented in three forest plots, grouped by outcome measure. Quality of life outcomes are presented in one forest plot. Other outcomes measured by questionnaires on which higher scores signify worse mental health symptomatology are presented together in a forest plot (stress, stigma, loneliness and other wellbeing measures), and the remaining outcomes in another forest plot (coping/self‐efficacy and social support). A final forest plot presents the overall SMD from meta‐analysis for each outcome measure.

For each of the nine outcome measure meta‐analyses, the percentage of between‐study heterogeneity was considered to be moderate if the *I* ^2^ statistic was 50–75%, and high if 75% and greater [14]. Higher levels of heterogeneity were accounted for by DerSimonian and Laird weighted random‐effects meta‐analysis that allows for between‐study heterogeneity.

### 2.7 Additional analyses for the outcome of depression

The possibility of publication bias in the meta‐analysis on depression (the most frequent outcome) was assessed using a funnel plot and Egger's test.

Sensitivity analyses were conducted to investigate overall SMD for depression across studies that had follow‐up time periods of: (i) up to 3 months; (ii) 4−6 months; and (iii) 7 or more months. The maximum time point over each period was included in the analysis.

A meta‐regression was conducted to investigate possible differences in intervention effectiveness on depression as there was some suggestion of borderline moderate between‐study heterogeneity (*I* ^2^ statistic = 40%) and the number of trials investigating depression exceeded 10. Nine pre‐defined intervention‐level factors were considered in the depression meta‐regression: (i) type of intervention (psychotherapeutic vs. not psychotherapeutic); (ii) intervention provider (psychologist vs. not psychologist); (iii) type of control group (standard care/standard care with waitlistvs. active control/enhanced standard care) ; (iv) mental health/wellbeing screening for study inclusion (yes vs. no); (v) mode of delivery (face‐to‐face vs. online/telephone); (vi) number of intervention sessions (1−8 vs. 9+); (vii) duration of each session in minutes (1−60 vs. 61+); (viii) overall length of intervention (from first to last session) in days (1−60 vs. 61+); and (ix) judgement of study quality (medium/low vs. high). Meta‐regression produces β coefficients that can be interpreted as unit changes in SMD.

Four supplementary meta‐regression analyses were conducted to compare intervention effectiveness on depression for:

- CBT versus non‐CBT interventions
- Motivational interviewing versus non‐motivational interviewing interventions
- Standard care controls versus not standard care, with the latter group including active control, enhanced standard care and waitlist controls
- Standard care controls versus not standard care, with the latter group including active control, enhanced standard care and “standard care with intentional support,” see footnote ^a^ under Table 4

Supplementary meta‐regression analyses (iii) and (iv) were conducted as it was considered likely that intervention effect size on depression would be dependent on the type of control group comparator, such that smaller effects would be reported in studies with an active control group and enhanced standard care, and potentially in those with waitlist controls. In a sensitivity analysis, the main meta‐analysis on depression was repeated after adjusting the effect size for all studies that did not use a standard care control group by the β coefficient found in meta‐regression for(a) standard care/standard care with waitlist versus not standard care (active control/enhanced care) controls and (b) standard care versus not standard care, with the latter group including active control, enhanced standard care and “standard care with intentional support” (see iv. above).

A final sensitivity analysis was carried out to investigate the effect of using Hedges’ *g*, which may account for small sample sizes [15], to calculate effect sizes in the meta‐analysis on depression.

This systematic review was performed according to the methods recommended by the Cochrane Collaboration [14]. All analyses were performed using STATA statistical software [16], and reported according to the PRISMA guidelines [17].

## 3 RESULTS

### 3.1 Identification and characteristics of included studies

Figure 1 presents the flow chart of study inclusion and exclusion. In total, 67 RCTs were included in this review; 66 were individually randomized parallel group‐controlled trials and one was a cluster randomized trial [18]. Sixty‐two studies contributed to at least one meta‐analysis as detailed below. Study characteristics are described in Table 3. Study sample size at baseline ranged from 23 to 463 participants who were randomized. Studies were from the following countries: the United States, Canada, the UK, Brazil, Italy, Spain, Australia, the Netherlands and Norway; the vast majority of studies were conducted in the United States (83.6%). Table 4 presents a summary with the frequency of key characteristics. Table 5 presents the frequency of specific interventions included in intervention classifications.

**Table 3:** Characteristics of the 67 included studies

Study	Country	Recruitment period	Population and number randomized	Mental health symptoms and/or well‐being screening for study inclusion	Intervention	Intervention classification	Control	Intervention provider^b^	Length of intervention (days)	Follow‐up time points (months)	Measures used
Barroso et al. [30]	U.S.	Information unavailable^a^	Women living with HIV (with internalized HIV stigma) *N* = 100	Internalized HIV Stigma Scale (score 40+)	Information (online), iPod touch with video including the experiences of being a women living with HIV	Supportive	iPod touch with nothing loaded on it	Computer	84	1, 3	CSES, RSES, IHSS
Barroso et al. [34]	U.S.	Information unavailable^a^	People living with HIV (score >5 HIV‐Related Fatigue Scale) *N* = 30	No	Cognitive Behavioral Stress Management app	Psychotherapeutic	Lifesum app (healthy lifestyle app)	Computer	70	1, 2, 5	STAI‐Trait, STAI‐State, BDI‐II, LES
Berko et al. [49]	U.S.	2020	People living with HIV (aged 50+ years with at least a minor degree of self‐reported loneliness) *N* = 214	3‐Item Loneliness (score 4+)	Mindfulness audio lessons (online)	General relaxation	Standard care with waitlist	Computer	25	1	GAD‐7, CES‐D‐10, 3IL
Blashill et al. [27]	U.S.	2013−2016	GBMSM living with HIV (with body image disturbance) *N* = 44	Body Dysmorphic Disorder modification of the Yale‐Brown Obsessive‐Compulsive Scale (score 16+)	CBT‐body image self‐care	Psychotherapeutic	Standard care with adherence support (enhanced standard care)	Healthcare worker	84	3, 6	MADRS
Bogart et al. [33]	U.S.	2018−2019	Latinx GBMSM living with HIV (not currently taking ART, missed at least one dose in the past month, or <2 HIV care visits in the past 12 months) *N* = 76	No	CBT and dialectical behaviour therapy, with psychoeducation	Multiple incl. psychotherapeutic	Standard care with waitlist	Peer	56	4, 7	Brief COPE dysfunctional, Brief COPE functional, IHSS
Bogart et al. [50]	U.S.	2018−2020	African American men and women living with HIV (missed at least one ART dose in the past month/detectable viral load in the last 6 months) *N* = 245	No	Motivational interviewing	Psychotherapeutic	Standard care	Peer	183	7, 13	IHSS
Bonato et al. [24]	Italy	2017	People living with HIV (with no medical conditions contraindicating physical exercise) *N* = 38	No	Physical exercise training using a smartphone app	Physical exercise	Physical exercise using hard copy training programme	Multiple not incl. psychologist (computer and exercise coach)	112	4	POMS
Brown et al. [51]	U.S.	2009	Women living with HIV *N* = 60	No	Stress management (online), with relaxation training	Multiple not incl. psychotherapeutic	Standard care with waitlist	Computer	1	1	CES‐D, CSES, BSI, POMS, HIV‐related Life‐Stressor Burden, PSS, Stress management knowledge
Brown et al. [18]	U.S.	2010−2012	Adolescents and young adults (aged 18–24 years with depressive symptoms) *N* = 4 sites (22 participants at intervention sites and 20 at control sites)^c^	Quick Inventory of Depressive Symptomatology‐Self‐Report (QIDS‐SR, score 7+)	Combination CBT and medication management algorithm	Multiple incl. psychotherapeutic	Standard care	Multiple incl. psychologist (psychologist and healthcare workers)	168	2, 3, 6	Remission from depression, QIDS‐SR, QIDS‐C, BHS, MSLSS
Brown et al. [37]	U.S.	2006−2007	GBMSM living with HIV *N* = 80	No	Information (group discussion), with stress management training	Multiple incl. psychotherapeutic	Standard care with waitlist	Peer	Information unavailable^a^	3	CSES, SPS, PSS
Carey et al. [52]	U.S.	2016−2017	People living with HIV (self‐reported ART non‐adherence/viral load >20 copies/ml, condomless sex or >1 sexual partner in the past 6 months, and depressive symptoms) *N* = 42	PHQ‐4 (score 2+)	Mindfulness training (tele)	General relaxation	Health coaching, with general health educational content (tele)	Trained facilitator	56	3	GAD‐7, PHQ‐9, PSS
Carrico et al. [53]	U.S.	2013−2017	GBMSM living with HIV (urine or hair sample reactive for methamphetamine) *N* = 110	No	Contingency management programme and positive affect intervention incl. mindfulness training	Multiple not incl. psychotherapeutic	Attention‐control using neutral writing exercise	Multiple not incl. psychologist (counsellor and trained facilitator)	35	3	DES positive affect, DES negative affect, FFMQ
Carroll et al. [41]	U.S.	2014−2017	People living with HIV *N* = 360	No	Access to personal health records via Apple iPod touch, training in eHealth, information, individual coaching session with a staff coach	Supportive	Standard care	Multiple not incl. psychologist (trained facilitator and peer)	Information unavailable^a^	12	PAM, MOS SF‐12
Cote et al. [54]	Canada	2012	People living with HIV *N* = 88	No	HIV treatment, virtual nursing assistance and education (web‐based intervention)	Supportive	Invited to consult list of websites with information on ART	Computer	28	6	MOS
Cunningham et al. [55]	U.S.	2012−2016	People being released from prison who are living with HIV *N* = 356	No	Peer navigation	Supportive	Standard care (with information on antiretrovirals	Peer	168	3, 6, 12	MOS SF‐12
DeMarco et al. [56]	U.S.	Information unavailable^a^	African American women living with HIV (age 40+ years) *N* = 111	No	Group writing and information	Supportive	Peer‐led support group	Peer	28	1, 6	STSS, HSS
Drozd et al. [57]	Norway	2011	People living with HIV (not infected via substance use) *N* = 67	No	Metacognitive therapy and positive psychology (online)	Psychotherapeutic	Standard care	Computer	35	1, 3	CES‐D, SWLS, PANAS, SWB
Flentje et al. [58]	U.S.	Information unavailable^a^	GBMSM living with HIV (with ≥1 occasion of drinking 5+ drinks in a single setting or of using an illicit substance in the past 3 months) *N* = 42	No	Psychoeducation and coping skills for minority stress	Supportive	Writing exercise focused on neutral content	Psychologist	63	2, 4	GAD‐7, PHQ‐9
Garcia et al. [59]	Canada	2016−2018	People living with HIV (with symptoms of poor sleep/mental health) *N* = 63	“One or more of the following symptoms: sleep problems, fatigue, pain, or anxiety or depression symptoms”	Autogenic training	General relaxation	Standard care	Healthcare worker	84	3, 6	PHQ‐9, STAI, PROQOL‐HIV
Giordano et al. [23]	U.S.	2010−2013	People living with HIV (who have been hospitalized; either newly diagnosed or out of care) *N* = 460	No	Peer mentoring	Supportive	Didactic sessions on safe sex and drug use	Peer	72	6	PHQ‐8, MOS SF‐36
Gonzalez‐Garcia et al. [60]	Spain	2011−2012	People living with HIV (diagnosed with HIV for ≥15 years, on ART for ≥5 years) *N* = 40	No	Mindfulness‐based cognitive therapy, with psychoeducation	Multiple incl. psychotherapeutic	Standard care	Psychologist	56	2, 5	BAI, BDI‐II, NHP, PSS
Gupta et al. [61]	U.S.	2015−2018	People living with HIV (HIV viral load <75c/ml and depressive symptoms) *N* = 54	PHQ‐9 (score 10+)	CBT (online)	Psychotherapeutic	Standard care with information about depression diagnosis and encouragement to follow‐up with primary care or HIV provider, and letter sent to primary provider	Computer	168	3, 6	PHQ‐9, HSC SCL‐20
Guy et al. [62]	U.S.	2011	GBMSM living with HIV (drank >14 drinks per week or ≥5 on a single occasion at least monthly) *N* = 180	No	Motivational interviewing	Psychotherapeutic	Standard care	Counsellor	14	3, 6, 12	CES‐D
Han et al. [63]	U.S.	2017	Women living with HIV (overdue for a Pap test) *N* = 123	No	Health literacy education and phone counselling	Supportive	Educational brochure related to cervical cancer	Trained facilitator	197	3, 6	PHQ‐9
Hart et al. [64]	Canada	2012−2017	GBMSM living with HIV (reporting condomless sex in the past 3 months) *N* = 183	No	Motivational interviewing, with information and group discussion	Multiple incl. psychotherapeutic	Standard care	Peer	56	3	Modified UCLA Loneliness, CES‐D
Hecht et al. [65]	U.S.	2005−2009	People living with HIV (not on ART and low likelihood of starting within 12 months) *N* = 177	No	Mindfulness‐based stress reduction	General relaxation	Education/HIV disease self‐management skills (group sessions)	Trained facilitator	56	3, 12	BDI‐II, DES negative affect, DES positive affect, PSS
Heckman et al. [39]	U.S.	2008−2010	People living with HIV (aged 50+ years with depressive symptoms) *N* = 361	GDS (score 10+)	Two intervention arms (i) Supportive expressive group therapy (tele) (ii) Coping effectiveness training (tele), with group discussion	Multiple incl. psychotherapeutic	Standard care	Psychologist	84	3, 4, 8	GDS
Heckman et al. [66]	U.S.	2010−2014	People living with HIV (with depressive symptoms) *N* = 167	PRIME‐MD	Interpersonal psychotherapy (tele)	Psychotherapeutic	Standard care	Psychologist	63	4	BDI‐II, IIPs, PSRS
Hersch et al. [26]	U.S.	2010−2011	People living with HIV (HIV viral load >48 copies/ml and detectable viral load) *N* = 168	No	ART adherence counselling (online), with information	Multiple incl. psychotherapeutic	Standard care with waitlist	Computer	Information unavailable^a^	3, 6, 9	SDS, PANAS, HIV/AIDS Stress Scale
Himelhoch et al. [40]	U.S.	2010−2011	People living with HIV (with depressive symptoms) *N* = 34	PHQ‐9 (score12+)	CBT (tele)	Psychotherapeutic	CBT (face‐to‐face)	Psychologist	98	2, 3	QID‐SR, HAM‐D
Jaggers et al. [67]	U.S.	Information unavailable^a^	People living with HIV *N* = 93	No	Exercise training	Physical exercise	Sedentary activity at the same exercise centre (e.g. reading a book, talking, watching TV)	Healthcare worker	42	1	POMS, SDS, PSS
Johnson et al. [68]	U.S.	2004	Mothers or primary care‐givers living with HIV (with children aged 4–12 years) *N* = 80	No	Parenting skills group intervention (information, with stress management)	Multiple incl. psychotherapeutic	Information on reducing health‐related stress, addressing the general stressors associated with living with HIV (group sessions)	Trained facilitator	42	6	PSI‐SF
Klein et al. [69]	U.S.	2011	African American women living with HIV *N* = 168	No	Information (watching a multimedia programme)	Supportive	Standard care, participants reviewed HIV education brochures	Computer	Information unavailable^a^	3	PSS
Lee et al. [70]	U.S.	2014−2015	People living with HIV (aged 45+ years and unemployed, retired or on disability, and reporting fatigue in past week) *N* = 53	No	Motivational interviewing (strategies for reducing fatigue)	Psychotherapeutic	Workbook focused on dietary strategies to reduce fatigue (designed to be credible, but with no empirical evidence)	Trained facilitator	1	1, 2, 3	HADS depression, HADS anxiety, DES
Lee et al. [71]	U.S.	2013−2017	GBMSM living with HIV (urine or hair sample reactive for methamphetamine) *N* = 110	No	Positive affect treatment, with online meditation exercises and contingency management programme (received i‐Pod)	Multiple incl. psychotherapeutic	Neutral writing exercise (received i‐Pod)	Multiple not incl. psychologist (counsellor and computer)	Information unavailable^a^	3, 6	DES
Lovejoy et al. [72]	U.S.	2009−2010	People living with HIV (aged 45+ years) *N* = 100	No	Motivational interviewing (tele) targeting sexual risk behaviour	Psychotherapeutic	Standard care	Clinicians enrolled in a doctoral programme in clinical psychology	28	3, 6	DASS
Magidson et al. [73]	U.S.	2009−2012	People living with HIV (receiving substance use treatment) *N* = 61	No	Behavioural activation and motivational interviewing to address substance use	Psychotherapeutic	Supportive counselling, avoiding behavioural activation or directive techniques (goal setting and activity planning)	Psychologist	30	3, 6, 12	BDI
Millard et al. [74]	Australia	2012−2013	GBMSM living with HIV *N* = 136	No	Group discussion (enhancing skills, confidence and ability, online and offline)	Supportive	Standard care	Peer	49	8, 12	HeiQ, PROQOL‐HIV, POSE
Moitra et al. [75]	U.S.	2014−2016	People living with HIV (new to care) *N* = 34	No	Acceptance‐based behaviour therapy (to facilitate the acceptance of HIV status)	Psychotherapeutic	Standard care	Psychologist	14	1, 3, 9	MSPSS
Moskowitz et al. [76]	U.S.	2008−2014	People living with HIV (newly diagnosed) *N* = 159	No	Behavioural and cognitive skills for increasing positive affect	Supportive	One‐to‐one contact with facilitator with no didactic portion or skills practice	Trained facilitator	98	5, 10, 15	CES‐D, DES negative affect, DES positive affect
Murphy et al. [77]	U.S.	2007−2008	Mothers living with HIV (who had not disclosed to their 6‐12 year olf child) *N* = 80	No	Information (preparing mothers for disclosure through behavioural exercises)	Supportive	Standard care	Trained facilitator	21	3, 6, 9	MHI, MOS SF‐36, GAD‐7
Murphy et al. [78]	U.S.	2011−2012	Mothers living with HIV, with child aged 6‐14 years *N* = 62	No	Information (parenting and self‐care, group discussion)	Supportive	Standard care	Researcher	28	3, 6, 12	GAD‐7, MHI
O'Cleirigh et al. [19]	U.S.	2013−2015	People living with HIV (smoking >5 cigarettes per day and motivated to quit) *N* = 53	No	CBT for smoking cessation plus nicotine replacement therapy	Psychotherapeutic	“Enhanced standard care” (weekly check‐in sessions plus nicotine replacement therapy)	Multiple incl. psychologist (doctoral‐level clinical psychology interns and postdoctoral fellows)	63	2, 6	STAI‐State, SIGH‐A, CES‐D, MADRS
Oliveira et al. [79]	Brazil	2014−2016	People living with HIV *N* = 23	No	Exercise training	Physical exercise	Recreational sessions composed of light activities such as stretching, recreational games and dancing	Trained facilitator	112	4	BAI, BDI, WHOQOL‐HIV
Ownby et al. [80]	U.S.		People living with HIV (aged 50+ years with mild neurocognitive disorder) *N* = 46	No	Computer‐delivered cognitive training and transcranial direct current stimulation	Supportive	Watching educational videos	Researcher	14	1	CES‐D, MOS SF‐36
Pyne et al. [81]	U.S.	2007−2008	People living with HIV (in VA healthcare system with depressive symptoms) *N* = 276	PHQ‐9 (score 10+)	Counselling and/or pharmacotherapy	Supportive	Standard care	Healthcare worker	Information unavailable^a^	6, 12	Depression free days, HSC SCL‐20, QWB, MOS SF‐12
Rao et al. [31]	U.S.	2013−2015	African American women living with HIV *N* = 239	No	Information to foster social support and reduce stigma (group discussion)	Supportive	Breast cancer education workshop	Multiple not incl. psychologist (social worker and peer)	2	4, 8, 12	SSCI
Roth et al. [25]	U.S.	2008−2009	People living with HIV *N* = 463	No	Behavioural change and motivational techniques	Psychotherapeutic	Standard care	Healthcare worker	Information unavailable^a^	12	MOS SF‐12
Saberi et al. [21]	U.S.	2018−2019	People living with HIV (aged 18–29 years) *N* = 50	No	Video‐counselling (including motivational interviewing and problem‐solving therapy) and text messaging focused on mental health, substance use and HIV care engagement	Psychotherapeutic	Standard care with waitlist	Multiple incl. psychologist (social worker and psychology fellow)	121	4, 8	GAD‐7, PHQ‐9, Brief Resilience Scale, PROMIS, Earnshaw and Chaudoir HIV stigma mechanisms
Safren et al. [82]	U.S.	2009−2012	People living with HIV (with depressive symptoms/antidepressant medication use) *N* = 240	CGI (score 2+) and current diagnosis of depression or antidepressant medication use	Two intervention arms (i) CBT and (ii) information & supportive psychotherapy, with adherence counselling	Multiple incl. psychotherapeutic	Standard care with a session of adherence counselling, a letter sent to the participant's treatment provider, and referral by the assessor for depression treatment if clinically indicated at follow‐up visits	Psychologist	77	4	CES‐D, CGI
Scharer et al. [83]	U.S.	2014−2015	African American men and women living with HIV (not taking or adhering to ART) *N* = 34	No	Motivational interviewing focused on ART adherence	Psychotherapeutic	Stress Reduction Skills Programme	Multiple not incl. psychologist (social worker and counsellor)	28	3, 6	BSI, MOS SF‐12
Schnall et al. [20]	U.S.	2016−2018	People living with HIV (experienced ≥2 HIV‐related symptoms in the past week) *N* = 80	No	Information (mHealth web‐app to assess symptoms, text message with tailored self‐care strategies and short video of strategies on app)	Supportive	Information (app without self‐care strategies)	Computer	84	3	PROMIS‐29 anxiety, PROMIS‐29 depression, MOS SF‐26
Schulte et al. [84]	U.S.		Mothers or primary care‐givers living with HIV (who had not disclosed their HIV status to their 6‐14 year old child) *N* = 176	No	Psychoeducation aiming to build self‐efficacy for disclosure	Supportive	Standard care with wait‐list (group‐based intervention delivered)	Trained facilitator	21	3, 9, 15	HIV health‐related anxiety, GAD‐7, CES‐D, MOS SF‐36
Scott et al. [85]	UK	2018−2019	People living with HIV (with depressive symptoms, and self‐reported neuropathic pain symptoms in their feet) *N* = 38	PHQ‐9 (score 10+ & ≤23)	Information (online), acceptance and commitment therapy with therapist feedback, and mindfulness practices	Multiple incl. psychotherapeutic	Standard care with waitlist	Multiple incl. psychologist (clinical psychologist and computer)	56	2, 5	PHQ‐9
Shah et al. [86]	U.S.	Information unavailable^a^	People living with HIV (aged 45+ years with functional limitations) *N* = 67	No	Physical activity counselling, with supportive telephone counselling sessions	Multiple not incl. psychotherapeutic	Standard care	Multiple incl. psychologist (physician, experienced mental health therapist and student)	84	3	BDI‐II, MOS SF‐36
Shuter et al. [87]	U.S.	2015−2016	People living with HIV (current cigarette smokers) *N* = 100	No	Information (mobile website with educational/motivational quit‐smoking sessions, and text messages and offer of nicotine patches)	Supportive	Standard care with brief structured advice to quit smoking, a self‐help brochure and an offer of nicotine patches	Computer	42	4	GAD‐7, CES‐D, Modified UCLA Loneliness, PSS‐4
Sibinga et al. [88]	U.S.	2015−2017	People living with HIV (aged 13–24 years old) *N* = 74	No	Mindfulness‐based stress reduction (MBSR)	General relaxation	Health education	Trained facilitator	56	3, 6, 12	MAAS, FFMQ
Simoni et al. [38]	U.S.	2009−2011	People living with HIV (Latinos of Mexican descent, sub‐optimally adherent to ART, with depressive symptoms) *N* = 253	BDI‐IA (score 10+)	CBT focused on adherence (and electronic pill box)	Psychotherapeutic	Standard care with notification letter to provider, and asked to return for brief monthly visits if had experienced problems with the pillbox or participation in the study.	Student	180	6, 9	MADRS, BDI‐IA
Stanton et al. [89]	U.S.	2014−2017	People living with HIV (current cigarette smokers showing readiness to quit) *N* = 442	No	Group therapy, with information, deep breathing techniques and nicotine patches	Multiple incl. psychotherapeutic	“Enhanced standard care” (standardized brief advice to quit, self‐help brochure and nicotine patches)	Multiple incl. psychologist (psychologist, social worker and peer)	42	4, 7	GAD‐7, DTS, Modified UCLA Loneliness, PSS‐4, PC‐PTSD
Stradling et al. [90]	UK	2013−2015	People living with HIV (with LDL cholesterol >3 mmol/l) *N* = 60	No	Motivational interviewing, with psychoeducation focused on Mediterranean style diet	Multiple incl. psychotherapeutic	Dietary advice promoting low saturated fat diet	Healthcare worker	183	6, 12	WEMWBS, EQ‐5D
Uebelacker et al. [91]	U.S.	2017−2020	People living with HIV (with depressive symptoms and self‐reporting chronic pain) *N* = 187	QIDS (score 9+)	Behavioural activation, coaching, with psychoeducation (including in‐person and telephone sessions)	Multiple incl. psychotherapeutic	Health education (including in‐person and telephone sessions)	Psychologist	21	3, 6, 9	Patient‐Reported Outcomes Measurement Information System, QIDS, MOS SF‐36
van Luenen et al. [43]	Netherlands	2015	People living with HIV (with depressive symptoms) *N* = 188	PHQ‐9 (score >4 and <20)	CBT (Internet‐based), with coaching, motivational interviewing, psychoeducation and telephone coaching	Multiple incl. psychotherapeutic	Standard care with waitlist, and with weekly callby a personal coach (monitored depressive symptoms and suicidal thoughts), and patients with severe depressive symptoms or suicidal ideation were referred to their general practitioner or HIV treatment centre	Multiple not incl. psychologist (coach and computer)	70	2, 3, 6	GAD‐7, CES‐D, PHQ‐9
Vance et al. [92]	U.S.	2017−2019	People living with HIV (aged 40+ years with HAND) *N* = 135	No	Cognitive training (online)	Supportive	Standard care	Multiple not incl. psychologist (trained facilitator and computer)	Information unavailable^a^	3	CES‐D, MOS‐HIV
Webel et al. [93]	U.S.	2008	Women living with HIV *N* = 89	No	Peer‐led group discussion focused on symptom management	Supportive	Received a copy of HIV symptom strategies manual	Peer	49	1, 3, 4	HAT‐QoL
Williams et al. [94]	U.S.	2007−2011	African American men living with HIV (engaged in unprotected anal/vaginal sex with a male and female partner in the past 3 months, and had a history of CSA) *N* = 117	No	Information focused on sexual risk reduction (group discussion), with stress management	Multiple incl. psychotherapeutic	Health promotion focused on general health and lifestyle	Peer	21	3, 6	BDI‐II, PDS
Wimberly et al. [95]	U.S.	2014−2016	People living with HIV (returning from jail, with problematic substance use or dependence) *N* = 75	No	Yoga (Hatha)	General relaxation	Standard care	Yoga teacher	84	3	PSS
Yigit et al. [22]	U.S.	2013−2017	People living with HIV (initiating HIV care) *N* = 372	No	Motivational interviewing to facilitate adjustment to living with HIV, supportive telephone calls	Multiple incl. psychotherapeutic	Standard care	Counsellor	336	11	Brief COPE, HSS, PHQ‐8

Abbreviations: BDI‐IA, Beck Depression Inventory; CGI, Clinical Global Impressions scale; CSA, childhood sexual abuse; GBMSM, gay, bisexual and other men who have sex with men; HAND, HIV‐associated neurocognitive disorder; PHQ, Patient Health Questionnaire; PLWH, people living with HIV; QIDS‐SR, Quick Inventory of Depressive Symptomatology‐Self‐Report.

^a^ Not reported in the article and no author response upon contact.

^b^ The category “Psychologist” includes psychotherapists and psychiatrists; “Healthcare worker” includes clinicians, nurses and social workers.

^c^ This was a cluster trial whereby sites were randomized not individual participants.

**Table 4:** Frequency of key characteristics of the 67 studies reviewed

	Psychosocial intervention studies among PLWH (*N* = 67) *n* (%)
**Country**	
U.S.	56 (83.6%)
Canada	3 (4.5%)
Australia	1 (1.5%)
Brazil	1 (1.5%)
UK	2 (3.0%)
Italy	1 (1.5%)
Spain	1 (1.5%)
The Netherlands	1 (1.5%)
Norway	1 (1.5%)
**Population (demographic group)**	
PLWH	42 (62.7%)
BAME PLWH	3 (4.5%)
GBMSM	8 (11.9%)
BAME GBMSM	2 (3.0%)
Women	4 (6.0%)
BAME women	3 (4.5%)
Mothers/primary care givers	4 (6.0%)
Prison population	1 (1.5%)
**Intervention classification**	
Psychotherapeutic	17 (25.4%)
Supportive	21 (31.3%)
General relaxation	6 (9.0%)
Physical exercise	3 (4.5%)
Multiple incl. psychotherapeutic	18 (26.9%)
Multiple not incl. psychotherapeutic	2 (3.0%)
**Control condition**	
Standard care^a^	27 (40.3%)
Standard care with waitlist^a^	9 (13.4%)
Enhanced standard care	3 (4.5%)
Active control	28 (41.8%)
**Intervention provider**	
Psychologist^b^	9 (13.4%)
Counsellor	2 (3.0%)
Healthcare worker^c^	6 (9.0%)
Peer	10 (14.9%)
Student	2 (3.0%)
Researcher	2 (3.0%)
Yoga teacher	1 (1.5%)
Trained facilitator	10 (14.9%)
Computer	11 (16.4%)
Multiple incl. psychologist	6 (9.0%)
Multiple not incl. psychologist	8 (11.9%)
**Intervention format**	
Face‐to‐face (individual)	30 (44.8%)
Face‐to‐face (group)	10 (14.9%)
Online/app/telephone^d^	27 (44.3%)
**Mental health screening**	
Yes	16 (23.9%)
No	51 (76.1%)

Abbreviations: BAME, Black, Asian and other ethnic minorities; GBMSM, gay, bisexual and other men who have sex with men; PLWH, people living with HIV.

^a^ Five studies described a standard care control group that received intentional support, for example letter to primary caregiver, phone calls, counselling.

^b^ Including psychotherapists and psychiatrists.

^c^ Including nurses and social workers.

^d^ Main component includes online intervention.

**Table 5:** Frequency of specific interventions included in intervention classifications

Intervention	Psychotherapeutic (*N* = 17) *n* (%)^a^	Supportive (*N* = 21) *n* (%)^a^	General relaxation (*N* = 6) *n* (%)^a^	Physical exercise (*N* = 3) *n* (%)^a^	Multiple incl. psychotherapeutic^b^ (*N* = 18) *n* (%)	Multiple not incl. psychotherapeutic^c^ (*N* = 2) *n* (%)
Cognitive behavioural therapy (CBT)	5 (29.4%)^d^	/	/	/	3 (16.7%)	/
Motivational interviewing (MI)	8 (47.1%)^e^	/	/	/	4 (22.2%)	/
Other talk therapies	2 (11.8%)	/	/	/	8 (44.4%)	/
Cognitive behavioural stress management	1 (5.9%)	/	/	/	4 (22.2%)	/
General counselling	/	3 (14.3%)	/	/	2 (11.1%)	2 (100.0%)
Peer support	/	3 (14.3%)	/	/		
Information/psychoeducation	/	13 (61.9%)^e^	/	/	13 (72.2%)	
Cognitive training	/	2 (9.5%)	/	/		
Mindfulness training	/	/	5 (83.3%)	/	4 (22.2%)	1 (50.0%)
Exercise training	/	/	1 (18.3%)^e^	3 (100.0%)		1 (50.0%)

^a^ Column percentages add up to 100%.

^b^ Seventeen studies included a psychotherapeutic intervention component as well as another type of intervention. Studies in this column are counted twice. Of note, one study included CBT, MI and psychoeducation [43] and is counted three times, and one study included talk therapy, psychoeducation and mindfulness practices [85] and is counted three times. The percentages shown do not add up to 100%.

^c^ Two studies included a supportive intervention component as well as another type of non‐psychotherapeutic intervention; physical exercise intervention and mindfulness training. Studies in this column are counted twice.

^d^ Not delivered by a professional psychologist.

^e^ Yoga.

### 3.2 Frequency of outcome measures

The questionnaire scales used to measure each outcome are presented in Table 2. Depression was the most common measure of mental health in this review. Forty‐five studies collected data on depressive symptoms, all of which were based on validated questionnaires. Mean scores (SD) were reported, or sent by authors upon request, in 40 studies, and are included in the meta‐analysis. Five studies did not present mean scores or proportions [19−23], and data were not available upon the author's request. Three of these studies contributed data to the meta‐analysis of other outcomes; quality of life [23], stigma [21, 22], social support/participation [21] and coping/self‐efficacy [21]. The remaining two studies did not contribute data to meta‐analyses in this review [19, 20].

Nineteen of the 22 studies that did not collect data on depressive symptoms contributed to the meta‐analysis of at least one other outcome measure. The remaining three studies did not contribute data to meta‐analyses in this review; median scores reported [24], no SD reported [25] and no data reported [26]. In total, 15 studies contributed data on anxiety symptoms to the meta‐analysis, 13 on stress, seven on stigma, four on loneliness, eight on coping/self‐efficacy, six on social support/participation, 19 on other measures of wellbeing, and for mental, physical, social and general quality of life; 12, 11, 6 and 7 studies, respectively, contributed data to the meta‐analyses.

### 3.3 Effect of psychosocial interventions on depression

**Figure 2:**
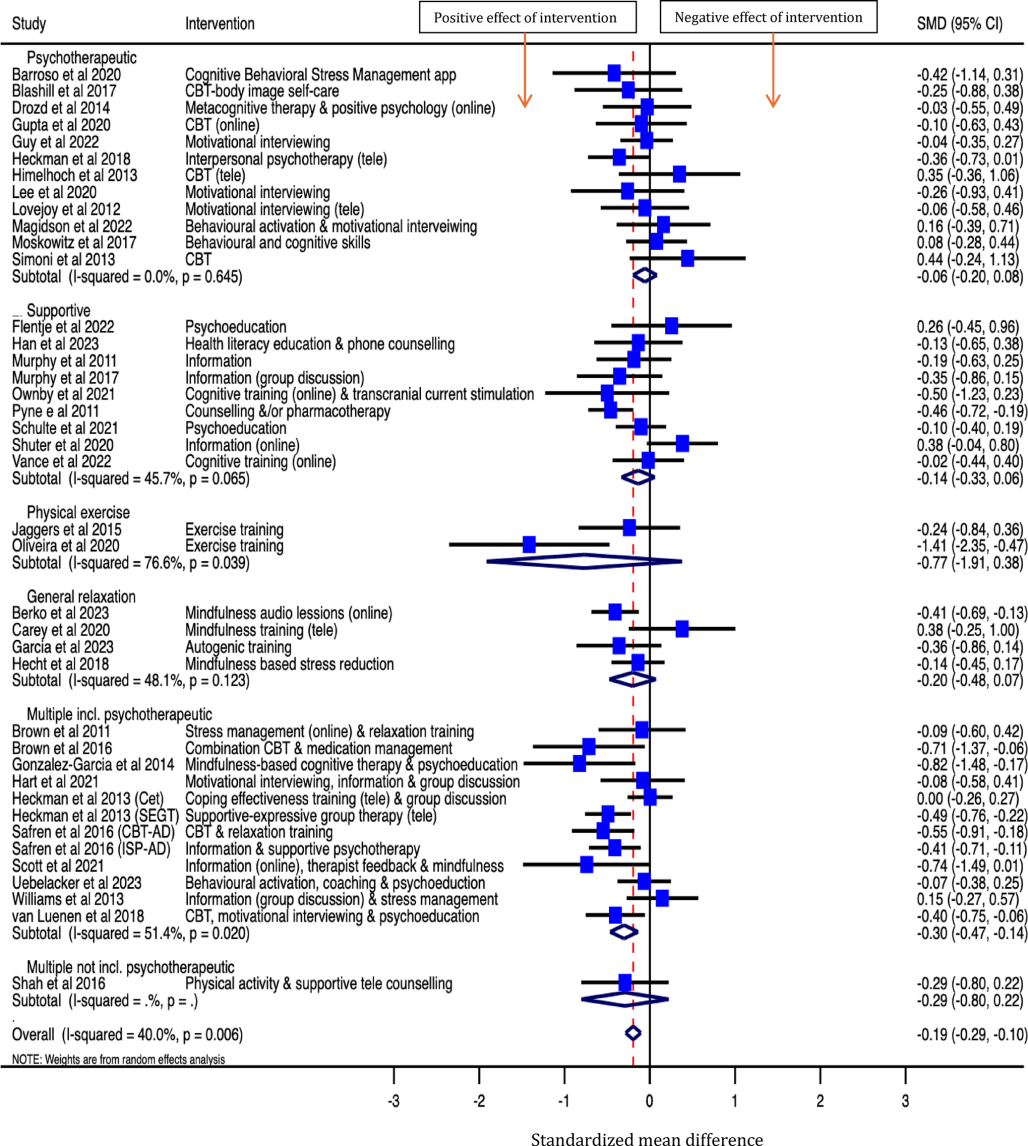
Effect of psychosocial interventions on symptoms of depression among people living with HIV (*N* = 40 interventions).

Higher scores on measures of depression relate to higher levels of symptomatology, therefore, negative effect estimates indicate that the intervention improved symptoms of depression compared to control. Across 40 studies, the overall SMD was −0.19 [95% CI: −0.29, −0.10], see Figure 2, indicating that the interventions reduced symptoms of depression compared to control. There was some suggestion of borderline moderate between‐study heterogeneity (*p* = 0.006; *I* ^2^ = 40.0%). Possible reasons for statistical heterogeneity between the results of the studies are discussed below. SMDs across studies ranged from −1.41 to 0.44. In sensitivity analysis, findings were very similar when using Hedges’ *g* to calculate effect sizes.

In a sensitivity analysis examining three different follow‐up time periods for the outcome of depression, the overall SMD was:

−0.19 [−0.27, −0.11]; *p*<0.001, across the 32 studies that had follow‐up periods of 0–3 months
−0.27 [−0.43, −0.10]; *p* = 0.001, across the 24 studies that had follow‐up periods of 4–6 months
−0.11 [−0.21, −0.02]; *p* = 0.024, across the 11 studies that had follow‐up periods of 7 or more months (8 months for 2 interventions, 9 months for 3 interventions, 12 months for 4 interventions and 15 months for 2 interventions)

Table 6 presents findings from the meta‐regression for the outcome of depression. Of studies that collected data on depression, 16 screened for poor mental health symptoms as an inclusion criterion, of which 12 screened for depressive symptoms specifically (the other four screened for: internalised HIV stigma; loneliness; symptoms of obsessive‐compulsive disorder; various symptoms including depression [27]). A greater effect on depression was found in studies that used mental health screening as an inclusion compared to those that did not (SMD −0.29 vs. −0.10, *p* = 0.023). There was also some evidence of a greater effect on depression for interventions that were conducted over 61 days or more (vs. ≤60 days) and that consisted of nine or more sessions (vs. <9) (*p*≤0.1). Results from the meta‐regression did not find evidence of a difference in intervention effect on depression for the other factors investigated: intervention type and provider, control group, mode of delivery, duration of each session and study quality (*p*≥0.2). The β coefficient for “standard care” versus “not standard care” control group was −0.13 and is utilized in a sensitivity meta‐analysis as presented below.

**Table 6:** Investigating key intervention characteristics as moderators of intervention effect on depression

	Meta‐analysis for depression measures	Unadjusted meta‐regression on depression measures	
	Overall SMD [95% CI]	β coefficient [95% CI]	*p‐*value
**Intervention type**			
Psychotherapeutic	−0.19 [−0.31, −0.08]	−0.03 [−0.24, 0.18]	*0.747*
Not psychotherapeutic^a^	−0.20 [−0.35, −0.04]	0	
**Intervention provider**			
Psychologist^b^	−0.26 [−0.42, −0.10]	−0.13 [−0.33, 0.08]	*0.220*
Not psychologist^c^	−0.16 [−0.27, −0.05]	0	
**Control group**			
Standard care^d^	−0.22 [−0.33, −0.11]	−0.12 [−0.33, 0.09]	*0.256*
Not standard care^e^	−0.14 [−0.31, 0.02]	0	
**Mental health/wellbeing screening for study inclusion**			
Yes	−0.29 [−0.42, −0.15]	−0.22 [−0.40, −0.03]	*0.023*
No	−0.10 [−0.21, 0.01]	0	
**Mode of delivery**			
Face‐to‐face	−0.18 [−0.31, −0.05]	0	*0.539*
Online/telephone	−0.23 [−0.35, −0.10]	−0.06 [−0.27, 0.14]	
**Number of intervention sessions** ^f^			
1−8	−0.11 [−0.22, −0.00]	1	*0.062*
9+	−0.29 [−0.48, −0.11]	−0.21 [−0.44, 0.01]	
**Duration of each session (minutes)** ^f^			
1−60	−0.19 [−0.34, −0.05]	0	*0.882*
61+	−0.21 [−0.38, −0.05]	0.02 [−0.23, 0.27]	
**Overall intervention length (days)** ^f^			
1−60	−0.12 [−0.23, −0.01]	0	*0.096*
61+	−0.26 [−0.41, −0.11]	−0.17 [−0.37, 0.03]	
**Study quality** ^g^			
High	−0.18 [−0.28, −0.08]	0	*0.442*
Medium/low	−0.24 [−0.43, −0.05]	−0.09 [−0.31, 0.14]	

Abbreviations: CI, confidence interval; SMD, standardized mean difference.

^a^ Supportive (help from others, without specific psychological technique), general relaxation, physical exercise.

^b^ Psychologist/psychotherapist/psychiatrist, counsellor.

^c^ Peer, computer, student, other healthcare worker, other trained facilitator and any other provider, for example researcher.

^d^ Includes waitlist control groups described as standard care by authors and those described by authors as “standard care” control group that received intentional support.

^e^ Active control groups and “enhanced standard care” controls.

^f^ Findings were similar when including this factor as a continuous variable in the meta‐regression.

^g^ Based on up to 12 risk of bias criteria assessed in each study.

In supplementary meta‐regression analyses, there was no difference in effectiveness between CBT versus non‐CBT interventions (β = −0.10 [−0.38, 0.17], *p* = 0.447), or motivational interviewing versus non‐motivational interviewing interventions (β = 0.15 [−0.16, 0.47], *p* = 0.333). Including waitlist control group studies in the “not standard care” control category did not impact on findings presented in Table 6. When the five “standard care with intentional support” studies were included in the “not standard care” control category, interventions with “standard care” control comparators had a greater effect on depressive symptoms than those with “not standard care” control groups (SMD −0.28 vs. −0.11); β = −0.19 [−0.39, −0.00], *p* = 0.049.

In a sensitivity meta‐analysis, after adjusting the intervention effect (SMD and 95% CI) on depression by (i) −0.12 and (ii) −0.19 (β coefficient for control group in meta‐regression, see Statistical methods), the overall SMD was:

−0.24 [−0.33, −0.15]
−0.29 [−0.38, −0.20]

**Figure 3:**
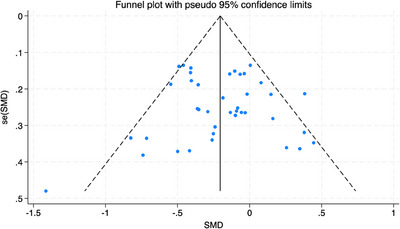
Investigating publication bias in 40 studies that contributed to the main depression analysis (Egger's test *p*‐value = 0.640).

There was no clear evidence of publication bias among studies included in the main meta‐analysis investigating intervention effectiveness on depression (Egger's test *p*‐value = 0.640), see Figure 3.

### 3.4 Effect of psychosocial interventions on other outcome measures

**Figure 4:**
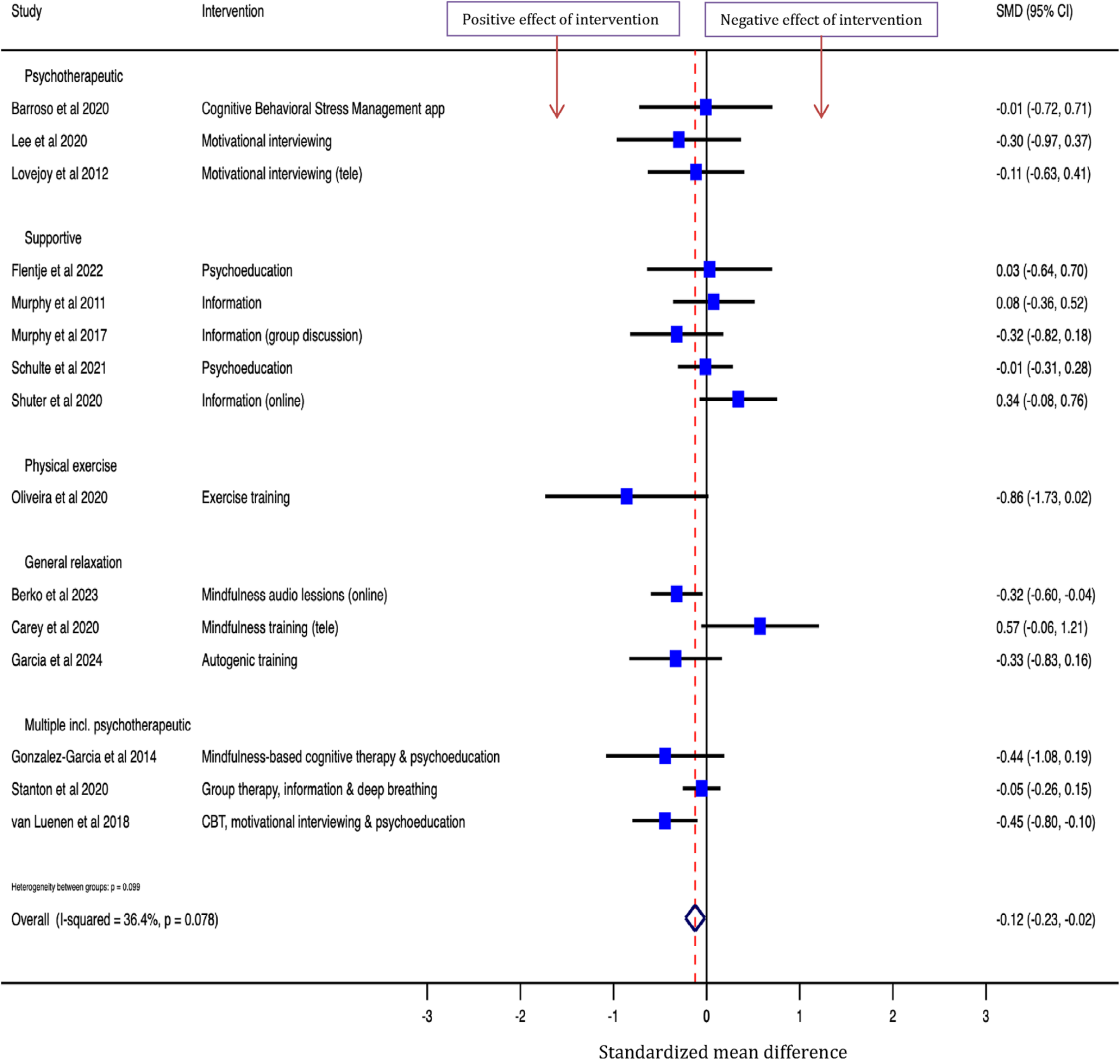
Effect of psychosocial interventions on symptoms of anxiety among people living with HIV (*N* = 15 interventions).

Across the 15 studies that collected data on anxiety, the interventions reduced symptoms of anxiety compared to control: overall SMD [95% CI] was −0.12 [−0.23, −0.02] (Figure 4).

**Figure 5:**
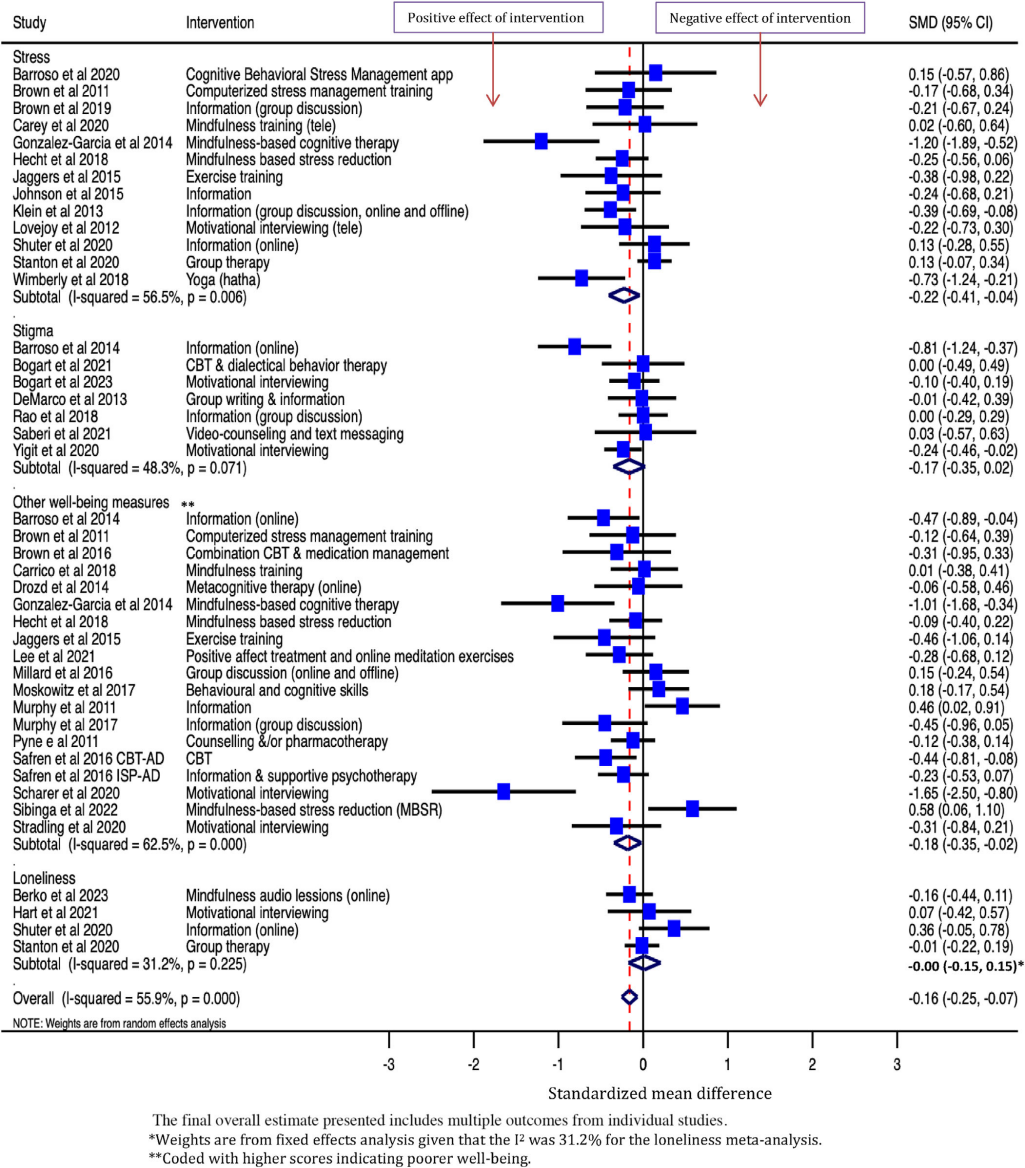
Effect of psychosocial interventions on stress (*N* = 13), stigma (*N* = 7), loneliness (*N* = 4) and other wellbeing measures (*N* = 19) among people living with HIV.

Across the 13 studies that collected data on stress, the interventions reduced stress compared to control: overall SMD was −0.22 [−0.41, −0.04]. Across the seven studies that collected data on stigma, the overall SMD was −0.17 [−0.35, 0.02]. Across the four studies that collected data on loneliness, the overall SMD was −0.001 [−0.15, 0.15]. Across the 19 studies that collected data on other wellbeing measures, the interventions reduced poor wellbeing compared to control: overall SMD was −0.18 [−0.35, −0.02] (Figure 5).

**Figure 6:**
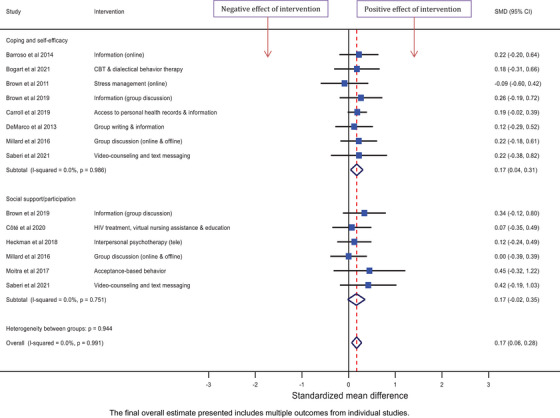
Effect of psychosocial interventions on measures of coping/self‐efficacy (*N* = 8) and social support (*N* = 6).

For the remaining measures, higher scores relate to greater wellbeing, therefore, positive effect sizes indicate that the intervention improved wellbeing. Across the eight studies that collected data on coping/self‐efficacy, the interventions increased coping/self‐efficacy compared to control with an overall SMD of 0.17 [0.04, 0.31]. Across the six studies that collected data on social support/participation, the overall SMD was 0.17 [−0.02, 0.35] (Figure 6).

**Figure 7:**
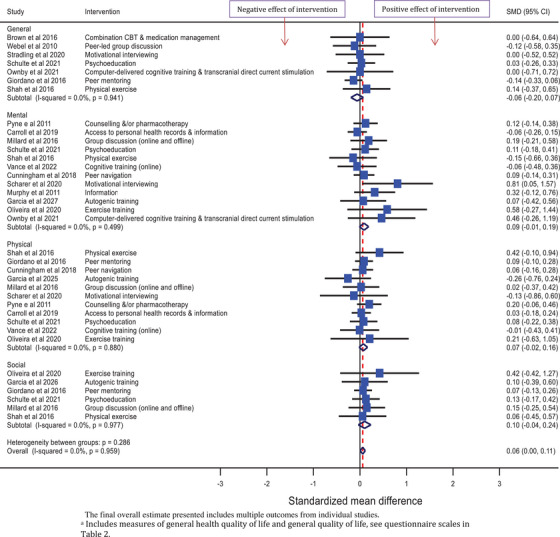
Effect of psychosocial interventions on measures of quality of life; general^a^ (*N* = 7), physical (*N* = 11), mental (*N* = 12) and social (*N* = 6).

Across the 11 studies that collected data on physical quality of life, the overall SMD was 0.07 [−0.02, 0.16]. Across the 12 studies that collected data on mental health quality of life, the overall SMD was 0.09 [−0.01, 0.19]. Across the six studies that collected data on quality of social life, the overall SMD was 0.10 [−0.04, 0.24], and across the seven studies that collected data on general quality of life, the overall SMD was −0.06 [−0.20, 0.07] (Figure 7).

### 3.5 Overall effects of psychosocial interventions on each outcome

**Figure 8:**
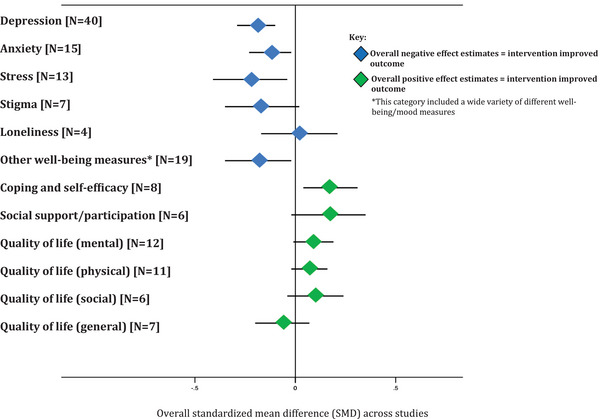
Overall effects of psychosocial interventions on each outcome measure.

Figure 8 presents the overall SMD of psychosocial interventions on the outcome categories from meta‐analyses. There is a clear pattern suggesting an overall positive effect of psychosocial interventions.

### 3.6 Quality of included studies

**Figure 9:**
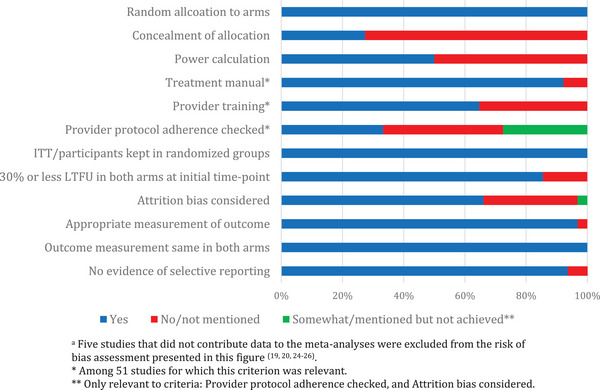
Risk of bias criteria assessed in 62^a^ studies included in analyses.

Quality assessment based on up to 12 criteria for determining possible risk of bias is presented in Figure 9. Of the 62 studies that contributed data in meta‐analyses, 66.1% were judged as being of high quality, 32.3% of medium quality and one study (1.6%) of potentially lower quality, see Table 7.

**Table 7:** Risk of bias criteria (up to 12)^a^ considered in 62^b^ studies included in analyses and percentage of criteria met

Study	1. Random	2. Conceal	3. Power	4. Manual	5. Training	6. Adherence	7. ITT	8.≤30% LTFU	9. Attrition	10. Measure‐ment	11. Outcome	12. Selective	Percentage criteria met (no. of criteria met/no. of relevant criteria)
Jaggers et al. 2015													41.7% (5/12)
Murphy et al. 2017													50% (6/12)
Johnson et al. 2015													50% (6/12)
Shah et al. 2016													58.3 (7/12)
Gonzalez‐Garcia et al. 2014													58.3% (7/12)
Oliveira et al. 2020													62.5% (7.5/12)
Pyne et al. 2011													62.5% (7.5/12)
Sibinga et al. 2022													66.7% (8/12)
Lee et al. 2020													66.7% (8/12)
Brown et al. 2019													66.7% (8/12)
Shuter et al. 2020				/	/	/							66.7% (6/9)
Lovejoy et al. 2012													66.7% (8/12)
Murphy et al. 2011													66.7% (8/12)
Han et al. 2023													66.7% (8/12)
Barroso et al. 2020				/	/	/							66.7% (6/9)
Webel et al. 2010													66.7% (8/12)
Flentje et al. 2022													66.7% (8/12)
Millard et al. 2016													66.7% (8/12)
Gupta et al. 2020				/	/	/							66.7% (6/9)
Scharer et al. 2020													70.8% (8.5/12)
Hecht et al. 2018													70.8% (8.5/12)
Lee et al. 2021													75% (9/12)
Williams et al. 2013													75% (9/12)
Rao et al. 2018													75% (9/12)
Giordano et al. 2016													75% (9/12)
Moitra et al. 2017													75% (9/12)
Bogart et al. 2021													75% (9/12)
DeMarco et al. 2013													75% (9/12)
Brown et al. 2011				/	/	/							77.8% (7/9)
Vance et al. 2022				/	/	/							77.8% (7/9)
Drozd et al. 2014				/	/	/							77.8% (7/9)
Garcia et al. 2023													83.3% (10/12)
Ownby et al. 2021													83.3% (10/12)
Simoni et al. 2013													83.3% (10/12)
Wimberly et al. 2018													83.3% (10/12)
Hart et al. 2021													83.3% (10/12)
Schulte et al. 2021													83.3% (10/12)
Carroll et al. 2019													83.3% (10/12)
Brown et al. 2016													83.3% (10/12)
Moskowitz et al. 2017													83.3% (10/12)
Saberi et al. 2021													86.4% (9.5/11)
Himelhoch et al. 2013													86.4% (9.5/11)
Heckman et al. 2013													86.4% (9.5/11)
Heckman et al. 2018													87.5% (10.5/12)
Magidson et al. 2022													87.5% (10.5/12)
Yigit et al. 2020													87.5% (10.5/12)
Carrico et al. 2018													87.5% (10.5/12)
Guy et al. 2022													87.5% (10.5/12)
Safren et al. 2016													87.5% (10.5/12)
van Luenen et al. 2018													87.5% (10.5/12)
Barroso et al. 2014				/	/	/							88.9% (8/9)
Klein et al. 2013				/	/	/							88.9% (8/9)
Scott et al. 2021				/	/	/							88.9% (8/9)
Cote et al. 2020				/	/	/							88.9% (8/9)
Berko et al. 2023				/	/	/							88.9% (8/9)
Blashill et al. 2017													91.7% (11/12)
Bogart et al. 2023													91.7% (11/12)
Carey et al. 2020													91.7% (11/12)
Stanton et al. 2020													100% (12/12)
Cunningham et al. 2018													100% (12/12)
Stradling et al. 2021													100% (12/12)
Uebelacker et al. 2023													100% (12/12)

^a^ Twelve risk of bias criteria include: 1. Random allocation to arms, 2. Concealment of allocation, 3. Power calculation, 4. Treatment manual, 5. Provider training, 6. Provider protocol adherence checked, 7. ITT/participants kept in randomized groups, 8. 30% or less LTFU in both arms at initial time point, 9. Attrition bias considered, 10. Appropriate measurement of outcome, 11. Outcome measurement same in both arms, 12. No evidence of selective reporting. If a criterium was considered to have been met (“yes”), it is coded in blue; if a criterium was not considered to have been met or was not mentioned (“no/not mentioned”), it is coded as red; if a criterium was considered to have been somewhat met or mentioned but not achieved (“somewhat” or “mentioned not achieved”), it is coded as green.

^b^ Five studies that did not contribute data to the meta‐analyses were excluded from the risk of bias assessment presented in this table [19, 20, 24, 25, 26].

## 4 DISCUSSION

There is evidence in this review of RCTs of an overall small to moderate positive effect of psychosocial interventions on reducing symptoms of depression, anxiety, stress and other measures of poor wellbeing, and increasing levels of self‐reported coping/self‐efficacy, over the short to medium‐term, among people living with HIV in high‐income countries. In meta‐regression relating to the depression outcome, interventions in studies that included only participants who met a threshold score for poor mental health symptoms had a significantly greater positive impact on depressive symptoms. This effect was as expected—the inclusion criteria ensured that the intervention was targeted at those with the greatest potential for benefit. If people with lower scores (better mental health) are included, there is less room for reduction in depression scores. There was also some evidence that longer interventions with a greater number of sessions were more beneficial than shorter ones. There was no evidence of a difference in intervention effectiveness for the other factors investigated, including the type of intervention (psychotherapeutic vs. other; CBT vs. other; motivational interveiwing vs. other), see the Limitations section for a discussion of control groups.

Psychosocial interventions also appeared to have a beneficial effect on stigma and social support/participation, with a similar magnitude of effect size; however, the overall SMDs were not statistically significant. Effect sizes were smaller, and statistically non‐significant, for measures of quality of life, and there was no difference found between study arms in reporting of loneliness, albeit data were available from only four studies.

These findings are in line with a systematic review of studies among people living with HIV, published between 1996 and 2014 (*n* = 62) [11]. The authors found an overall small positive effect of psychosocial interventions on mental health (depression, anxiety, quality of life and other psychological wellbeing measures) compared to a control condition (Hedges’ *g* = 0.19 [95% CI: 0.13, 0.25], *p* = 0.001). This effect was also found to lessen over time. Greater intervention effects on depression were found for psychologist‐delivered interventions, in studies with mental health as a primary focus of the intervention, and as in our review, in studies with depressive symptoms as an inclusion criterion. The current review extends that conducted previously by including recent data and separating outcome data into specific constructs, that is depression, anxiety, coping and self‐efficacy, stress, stigma, loneliness, social support/participation and quality of life (mental, physical, social and general). The current review also incorporated a broader range of psychosocial interventions by including physical intervention studies (e.g. exercise training) and by not excluding articles with no mention of mental health/wellbeing/quality of life in the title, abstract or key words. Findings from this review are specific to high‐income countries.

Depression was the outcome measure most frequently investigated across psychosocial intervention studies in this review (*N* = 45 interventions). There was no evidence found for a difference in effect on depression between the various psychosocial (or psychotherapeutic) intervention types. Recognition in the literature that different types of psychological therapy often achieve similar results when compared to control [11, 28] has given rise to the hypothesis that there are important factors common to all forms of therapy, specifically the therapeutic alliance—the collaborative relationship between patient and therapist [29]. Experience and competence in mental healthcare is considered vital to the therapeutic alliance [29]. Most studies included in this meta‐analysis (51 out of 62) involved some form of help from another person (as opposed to being exclusively self‐help/online), but it was not possible to assess the magnitude of effect size according to the amount of experience of the provider. Psychologist (vs. non‐psychologist) delivered interventions were associated with a greater reduction in depressive symptoms, although a statistically significant difference was not found, but there might have been a lack of power to detect a difference.

A small number of psychosocial intervention studies (*N* = 7) were identified in the current review that included stigma as an outcome. The overall effect size for stigma in the meta‐analysis was in line with estimates for other outcomes but was not statistically significant. Only three of the seven studies focused on stigma as the primary endpoint; interventions were provision of information online [30], motivational interviewing [22] and group discussions [31]. Historically, HIV research typically acknowledged Goffman in reference to stigmatization, and stigma came to be seen as something inherent in a person living with HIV [32]. Most studies responding to HIV‐related stigma have delivered information to segments of the general population in an effort to change attitudes to what was perceived as an undesirable attribute. More recently, however, stigma has been reframed as a constantly changing (and often resisted) social process [32]. HIV‐related stigmatization may, therefore, be thought of as a function of social inequality in society, in which relations of power and control diminish certain population groups and elevate others. The stress of intersectional stigma, heightened discrimination based on combinations of gender, sexuality and ethnicity, is increasingly acknowledged in HIV research [33], as is the importance of internalized HIV stigma, the process by which people internalize or endorse society's negative beliefs about HIV [4]. The ways in which stigmatization and discrimination have been addressed in intervention studies among people living with HIV may have been limited by past approaches to conceptualizing and investigating stigma.

The number of studies in this review that investigated stress as an outcome was also relatively small (*N* = 13), with only one investigating HIV‐related stress [34]. There was a small overall reduction in stress in the meta‐analysis, which was statistically significant. Very few studies in this review (*N* = 4) included loneliness as an outcome, even though loneliness represents a critical concern for people living with chronic health conditions including HIV [35] and has been linked to disruptions in immune function, increased inflammation and altered glucocorticoid release, with implications for poor health outcomes [36]. Six studies had outcomes related to social support/participation, across which there was a small non‐significant positive effect. Several interventions in this review (15%) included a group‐based element, providing a possible forum for participants to expand their social networks and garner support from others with shared experience. It has been suggested that the follow‐up time for such studies is too short to capture potential improvements in social support/participation that develop over time [37].

In this review, half of the studies (53.7%) included a standard care control group, the remainder analysed an active or enhanced standard care control group who were given some form of intervention. There may be ethical considerations when randomizing individuals with unmet psychological need to standard of care, possibly explaining the use of active controls. However, comparison with an active control group is likely to dilute the effectiveness of the intervention, leading to more conservative estimates of intervention effect. In addition, some studies in this review described their control group as standard care, but there was some indication that participants may have received care above and beyond that which is typically given. Studies with waitlist control groups were also described as standard care, but the anticipation of intervention may affect participants’ mental health and wellbeing. This has implications for the categorization of control groups and may suggest that intervention effect sizes tend to be underestimated. In a supplementary meta‐regression analysis, interventions that were compared to standard care controls had a significantly greater effect on depression versus interventions compared to not standard care (including enhanced standard care) control groups (when the latter category included “standard care with intentional support” controls). After adjusting for the effect of control group type, a sensitivity meta‐analysis revealed that the overall SMD for depression may be up to −0.29 [−0.38, −0.20], suggesting a small to moderate overall effect of psychosocial intervention on depression.

Furthermore, there may be limitations to combining effect estimates across different measures within a particular outcome category. The constructs of depression and anxiety are relatively well defined, and arguably consistently captured across the numerous questionnaires used to measure these conditions, at least in terms of relative score, if not absolute level. However, other constructs investigated in this review, such as stress, stigma and quality of life, may be interpreted differently across questionnaires, affecting the overall findings in the meta‐analysis. For instance, the questionnaires used to measure stigma captured aspects of internalized, enacted and/or anticipated stigma. The questionnaires used to measure stress included stress related to life events, every day emotional states or parenting. The questionnaires used to measure general quality of life were particularly heterogeneous, capturing the quality of one's general health or quality of life in general. Questionnaire scales are presented in full in Table 2.

There may be a lack of generalizability of findings in this meta‐analysis given that all included studies utilized convenience sampling for participant recruitment. Further, 84% of studies were conducted in the United States, and findings may be less applicable to other high‐income country settings including the UK. We did not consider interventions conducted in low/middle‐income countries; however, some interventions that have been evaluated in low‐income settings may also be relevant and applicable to high‐income settings. Future systematic reviews of psychosocial interventions in people living with HIV may wish to include studies considered outside the scope of this review such as, observational studies or studies including adolescent participants.

A sensitivity analysis in this review suggested that interventions were less effective overall in reducing depressive symptoms over the longer‐term. A key theme discussed across studies was the need for booster sessions to retain positive effects within one to 6 months after intervention completion. It was suggested in one study that to sustain any beneficial intervention effect regarding stigma, ongoing social support via regular peer support groups, even in a clinical setting, would be needed, as stigma may still be experienced in families, communities and places of work [38]. Findings from the meta‐regression in this review also suggest that longer interventions (≥61 days), with a greater number of sessions (≥9), tended to be more beneficial.

Across studies in this review, there were conflicting strategies used in terms of exclusion of participants based on psychiatric conditions. Some studies stated that individuals were not excluded on the basis of psychiatric history in order to assemble a more externally valid sample [39], whereas others argued it was potentially dangerous and unethical to include people with severe psychiatric conditions in RCTs [40].

In the UK, health coaching (to build patient knowledge, skills and confidence) is being rolled out on the National Health Service (NHS) for chronic conditions and there has been a big investment in social prescribing in primary care.  In the current review, very few interventions included “coaching,” and none included “social prescribing.” Four interventions included a component described as coaching [25, 41, 42, 43]. In one study, the control condition consisted of health coaching [44]. However, some studies may have included equivalent interventions that were labelled differently. Further research is needed to investigate the effectiveness of health coaching and social prescribing for people living with HIV.

## 5 CONCLUSIONS

Current evidence from RCTs in high‐income settings suggests that people living with HIV may benefit from psychosocial interventions, with evidence that these interventions had a beneficial impact on symptoms of depression, anxiety, stress, self‐efficacy/coping and other measures of wellbeing. Interventions were most effective in reducing depression when targeted only at those who met a threshold mental health score at baseline. Pooled effect estimates were small, or small to moderate in this review, with an indication that the widespread use of active control groups results in an underestimation of true effect size. Psychosocial interventions may also improve other measures of wellbeing and quality of life, although the overall findings in this review were not statistically significant, and very few studies investigated stigma and loneliness outcomes. The specific type of psychosocial intervention does not appear to determine effectiveness on depression, in line with previous research. Future RCTs could consider the use of mental health/wellbeing screening as an eligibility criterion, the type of control group comparator, and the length and intensity of the intervention, as these factors may influence the effect size.

## COMPETING INTERESTS

No potential conflict of interest was reported by the authors.

## AUTHORS’ CONTRIBUTIONS

FCL and AR conceived the review, and FCL, AR, ARM, JS, ANP, LS, SVL, NG and VK developed the search strategy. ARM and JS carried out the systematic searches. FCL, VC, ARM, JS and FN developed the data extraction protocol and designed the meta‐analysis. ARM, JS and SMR extracted the data. ARM drafted the manuscript and conducted all analyses. All authors contributed to data interpretation, writing, revision and approval of the final manuscript.

## FUNDING

This study represents independent research funded by the National Institute for Health Research (Programme Grants for Applied Research, A person‐centred Needs Informed model of Care for people with HIV [NICHE], to improve wellbeing, mental health and reduce socio‐economic disadvantages and stigma, NIHR202038).

## DISCLAIMER

The views expressed are those of the authors and not necessarily those of the NHS, the National Institute for Health Research or the Department of Health and Social Care.

## Data Availability

All data are presented in the figures.
